# Dose–volume histogram analysis and clinical evaluation of knowledge-based plans with manual objective constraints for pharyngeal cancer

**DOI:** 10.1093/jrr/rraa021

**Published:** 2020-04-24

**Authors:** Takuya Uehara, Hajime Monzen, Mikoto Tamura, Kazuki Ishikawa, Hiroshi Doi, Yasumasa Nishimura

**Affiliations:** 1 Department of Radiation Oncology, Kindai University Faculty of Medicine, Osaka-Sayama, Osaka, Japan; 2 Department of Medical Physics, Graduate School of Medical Sciences, Kindai University, Osaka-Sayama, Osaka, Japan

**Keywords:** intensity-modulated radiation therapy, knowledge-based planning, nasopharyngeal cancer, oropharyngeal cancer, volumetric-modulated arc therapy

## Abstract

The present study aimed to evaluate whether knowledge-based plans (KBP) from a single optimization could be used clinically, and to compare dose–volume histogram (DVH) parameters and plan quality between KBP with (KBP_CONST_) and without (KBP_ORIG_) manual objective constraints and clinical manual optimized (CMO) plans for pharyngeal cancer. KBPs were produced from a system trained on clinical plans from 55 patients with pharyngeal cancer who had undergone intensity-modulated radiation therapy or volumetric-modulated arc therapy (VMAT). For another 15 patients, DVH parameters of KBP_CONST_ and KBP_ORIG_ from a single optimization were compared with CMO plans with respect to the planning target volume (D_98%_, D_50%_, D_2%_), brainstem maximum dose (D_max_), spinal cord D_max_, parotid gland median and mean dose (D_med_ and D_mean_), monitor units and modulation complexity score for VMAT. The D_max_ of spinal cord and brainstem and the D_med_ and D_mean_ of ipsilateral parotid glands were unacceptably high for KBP_ORIG_, although the KBP_CONST_ DVH parameters met our goal for most patients. KBP_CONST_ and CMO plans produced comparable DVH parameters. The monitor units of KBP_CONST_ were significantly lower than those of the CMO plans (*P* < 0.001). Dose distribution of the KBP_CONST_ was better than or comparable to that of the CMO plans for 13 (87%) of the 15 patients. In conclusion, KBP_ORIG_ was found to be clinically unacceptable, while KBP_CONST_ from a single optimization was comparable or superior to CMO plans for most patients with head and neck cancer.

## INTRODUCTION

The clinical use of intensity-modulated radiation therapy (IMRT) and volumetric-modulated arc therapy (VMAT) represents significant advances in radiation therapy. IMRT is effective, especially in patients with head and neck cancer, because the clinical target volumes (CTV) generally border organs at risk (OARs) such as the salivary glands, brainstem and spinal cord [1]. However, several clinical limitations are associated with the planning of IMRT for head and neck cancer. These include (i) the time-consuming optimization process, (ii) the achievable dose–volume histogram (DVH) being unknown at the time of optimization, and (iii) the dependence of plan quality on the planners’ (or institutions’) experience and skills [2–11].

One approach that can be used to improve speed and efficiency and reduce variability in treatment planning is the so-called knowledge-based plans (KBP) approach. KBP are defined as any approach that directly uses prior experience to either predict an achievable dose for a new patient or to derive a better starting point for optimization by a planner [12]. Rapidplan™, a commercial KBP tool, is integrated into the Eclipse (Varian Medical Systems, Palo Alto, USA) treatment planning system (TPS) and is a machine-learning tool that uses best practices from previous treatment plans to create knowledge-based models for the treatment of new patients. Comparisons of KBP with clinical manual optimized (CMO) plans for prostate cancer have demonstrated that KBP can lead to clinically acceptable DVHs [2, 5, 6, 12–14].

Fewer reports have compared KBP with CMO plans in head and neck cancer than in prostate cancer [12], although most investigators have demonstrated that KBP are advantageous for DVH analysis of head and neck cancer [3, 4, 7, 8, 15]. We consider it is clinically important to accurately establish the usefulness of KBP based on various reports. Several investigators did not set objective constraints manually for OARs [3, 4, 7, 16]. Wang *et al*. did not separate the training and validation sets [8], while Krayenbuehl *et al*. did not evaluate the DVH of brainstem [15]. In addition, Chang *et al*. reported that re-optimization of KBP was required for nasopharyngeal cancer (NPC), because the KBP DVH parameters for neurological structures were poorer than those of CMO plans [11]. We therefore sought to establish the advantage of KBP for head and neck cancer with or without objective constraints forOARs.

The present study aimed to evaluate whether KBP from a single optimization could be used in clinical settings by adding objective constraints manually. Thus, in the present study, KBP without manual setting of objective constraints (KBP_ORIG_) and those with objective constraints (KBP_CONST_) were compared with CMO plans for patients with NPC or oropharyngeal cancer (OPC).

## MATERIALS AND METHODS

### Patient selection and contouring

A training set of 55 clinical plans for NPC or OPC treated by IMRT or VMAT between 2014 and 2018 was used to train the KBP. Treatment plans were VMAT in 12 cases (22%) and IMRT in 43 cases (78%). As our institution adopted the VMAT technique from 2016, the number of VMAT plans was small in the training set. Staging was performed according to the tumor-node-metastasis (TNM) classification system (7th edition) of the International Union Against Cancer (UICC). As a validation set, further 15 clinical VMAT plans between 2017 and 2018 were used to compare the single-optimization KBP with CMO plans. Since the patients were recruited in a consecutive manner, the number of NPC patients was low in the validation set. As all patients were treated with whole neck irradiation, treatment plans were similar for NPC and OPC. All CMO plans were used clinically for the 15 patients. The characteristics of patients whose plans were used for training and validation are summarized in Table 1.

**Table 1:** Characteristics of patients in the training and validationsets

	Training set (*n* = 55)	Validation set (*n* = 15)
Age, years (median; range)	68; 28–89	61; 40–78
Male/female	36 (66%)/19 (34%)	14 (93%)/1 (7%)
Primary sites		
Nasopharyngeal cancer	19 (35%)	1 (7%)
Oropharyngeal cancer	36 (65%)	14 (93%)
TNM stage (7th edition)		
I	7 (13%)	0 (0%)
II	8 (15%)	0 (0%)
III	11 (20%)	3 (20%)
IVA	20 (36%)	8 (53%)
IVB	7 (13%)	4 (27%)
IVC	2 (3%)	0 (0%)

All patients were immobilized and thermoplastic masks were used to cover the head, neck and shoulders (Type-S thermoplastic-based system; MED-TEC, Orange City, IA, USA). Contrast-enhanced computed tomography (CT) scans for treatment planning were obtained at 2-mm slice intervals from the head to the aortic arch [17, 18]. CTV encompassed a 5.0–10.0 mm margin with appropriate anatomical correction around the gross tumor volume. The prophylactic nodal CTV was defined and delineated according to the Danish Head and Neck Cancer Group, the European Organization for Research and Treatment of Cancer, the French Group of Radiation Oncology for Head and Neck Cancer, the French Head and Neck Cancer Group, the National Cancer Institute of Canada and the Radiation Therapy Oncology Group consensus guidelines [19]. The nasopharyngeal or oropharyngeal region, the bilateral level II–IV nodes and the retropharyngeal nodes were included in the initial CTV [20]. Submandibular lymph nodes (level Ib) were only included in the CTV where their involvement was suspected. Margins of 3.0–4.0 mm were added to the CTVs to determine the planning target volume (PTV), thus allowing for errors associated with treatment set-up and internal organ motion error [21]. The brain, brainstem, inner ears, eyes, larynx, lens, pharyngeal constrictor muscles, optic nerves, chiasm, mandible bone, oral cavity, spinal cord, parotid glands and thyroids were included as OARs at our institution. A 3.0-mm margin was added to the spinal cord as planning organ at risk volume (PRV). No margin was added to the parotid glands, and parotid glands minus PTV were used for treatment planning whereas DVH parameters were evaluated for each parotid gland. The treatment planning CTs and delineated structures used for the CMO plans were also applied to KBP, using the same methods.

### Dose prescription and treatment planning

A two-step IMRT method is used at our institution instead of the simultaneous integrated boost method [1, 17, 18]. In the initial planning, the prescribed dose was 70 Gy in 35 fractions to the initial PTV. After whole-neck radiotherapy of 44–50 Gy in 22–25 fractions had been delivered in the initial plans, a boost plan was administered to the high-risk CTV up to a total dose of 70 Gy in 35 fractions. In the present study, the KBP and CMO plans were compared with the initial plans for the whole-neck region. The prescribed dose was normalized to the dose of 70 Gy to 95% of the PTV [18]. Our goals and acceptable criteria for DVH parameters are shown in Table 2.

**Table 2:** Our DVH goal and acceptable criteria for PTV andOARs

	Parameter	DVH goal	Acceptable criteria
PTV^a^	D_98%_	>93%	>90%
	D_50%_	<105%	<107%
	D_2%_	<120%	<125%
Spinal cord	D_max_	<50.0 Gy	<54.0 Gy
Brainstem	D_max_	<54.0 Gy	<64.0 Gy
Parotid gland^b^	D_med_	<20.0 Gy	<24.0 Gy
	D_mean_	<26.0 Gy	<30.0 Gy

^a^100% doses were set to 70 Gy.

^b^At least one parotid gland.

All IMRT plans in the training set were created using Eclipse ver. 7.3.10 (Varian Medical Systems, Palo Alto, USA) and optimized with a dose volume optimizer. These IMRT plans were delivered using a dynamic multileaf collimation from one of two linear accelerators (Clinac 600C or Clinac 21EX; Varian Medical Systems, Palo Alto, USA) equipped with a 40-leaf dynamic multileaf collimator. Beam energies of 4 or 6 MV X-rays wereused.

All VMAT plans for the training and validation sets were created using an Eclipse TPS ver. 13.6 with 6 or 10 MV photon beams. Two full arcs of VMAT were applied (gantry angle: 181–179° clockwise and 179–181° counterclockwise; collimator angles: 5 and 85°, or 30 and 330°). The control point spacing was 2° of angular separation. All VMAT plans were optimized with a photon optimizer and calculated using the Varian analytic anisotropic algorithm and an Eclipse TPS for a TrueBeam (Varian Medical Systems, Palo Alto, USA) with a Millennium 120 multileaf collimator.

### Model configuration for the knowledge-based optimized plans

The KBP model configuration and training process is described in previous reports [2, 14, 22]. In the KBP optimization process, optimization objectives named ‘line objectives’ were upper objectives placed on line and along the inferior DVH prediction boundary for OARs, and priority values were automatically generated (KBP_ORIG_). In addition, we could modify objective constraints for OARs manually after generating objective constraints automatically (KBP_CONST_). For both KBP_ORIG_ and KBP_CONST_, objective constraints for the PTV needed to be manually set. In this study, the upper and lower objectives for the PTV were set at 70–70.7 Gy and 67.9–69.3 Gy, respectively for both KBP_ORIG_ and KBP_CONST_. Most previous investigators used only automatically generated objective constraints for OARs and compared the DVH parameters between the KBP and CMO plans [2, 4, 6, 7, 11, 13–15, 22, 23]. Alternatively, Kamima *et al*. reported that KBP with manual objective constraints resulted in better OAR sparing compared with KBP without manual objective constraints for single optimization, especially for the brainstem and spinal cord [24]. In the present study, we evaluated the DVH parameters for KBP_ORIG_ and then manually added the objective constraints for OARs to the optimization of the KBP_CONST_, whereas we did not change the objective constraints for PTV. Details of the objective constraints for KBP_CONST_ are summarized in Table 3. Additionally, the priority of normal tissue objectives (NTO) was set to 200 for KBP_CONST_, although the default priority of 100 was used for KBP_ORIG_. At our institution, the priority value of NTO was set at the same value as that of PTV, thus avoiding the creation of a high-dose region on the posterior side of the patient. For 14 patients with OPC and one with NPC, the KBP_ORIG_ or KBP_CONST_ were created using a single optimization without any planner intervention [2].

**Table 3:** Objective constraints for KBP_CONST_

		Vol (%)	Dose (Gy)	Priority
PTV	Upper	0	70.7	175
	Upper	0	70.0	200
	Lower	100	69.3	200
	Lower	100	67.9	150
Brain	Upper	0	63.0	50
	Line	Automatically generated		
Brainstem	Upper	0	44.1	200
	Line	Automatically generated		
Left inner ear	Upper	0	56.0	50
	Line	Automatically generated		
Right inner ear	Upper	0	56.0	50
	Line	Automatically generated		
Larynx	Mean		31.5	Automatically generated
	Line	Automatically generated		
Mandible	Upper	50	42.0	50
	Line	Automatically generated		
Pharyngeal constrictor muscle	Upper	0	50.4	60
	Mean		35.0	60
	Line	Automatically generated		
Oral cavity	Upper	50	30.0	60
	Line	Automatically generated		
Left parotid gland minus PTV	Upper	0	49.0	80
	Mean		18.0	100
	Line	Automatically generated		
Right parotid gland minus PTV	Upper	0	49.0	80
	Mean		18.0	100
	Line	Automatically generated		
Spinal cord	Upper	0	42.0	200
	Upper	30	30.1	100
	Upper	50	24.5	100
	Line	Automatically generated		
Thyroid	Line	Automatically generated		
NTO				200

### DVH analysis and plan quality evaluation

The DVH parameters of the KBP_ORIG_, KBP_CONST_ and CMO plans of the 15 patients of the validation set were compared in terms of the D_98%_, D_50%_ and D_2%_ for the PTV, where D_98%_, D_50%_ and D_2%_ are the doses received by 98, 50 and 2% of the PTV, respectively. In the present study, DVH parameters of the spinal cord, brainstem and parotid glands were evaluated, because these three OARs were the most important for IMRT plans: D_max_ (maximum dose) of the spinal cord and brainstem, D_med_ (median dose) and D_mean_ (mean dose) of the parotid glands. Moreover, the number of monitor units (MUs) and modulation complexity score for VMAT (MCSv) were evaluated[25, 26]. The ipsilateral and contralateral parotid glands were evaluated separately [3]. At our institution, treatment plans were created and evaluated according to the Japan Clinical Oncology Group (JCOG) 1015 protocol (Table 2) [27]. Paired *t*-tests were used to identify differences between KBP_ORIG_, KBP_CONST_ and CMO plans, with *P*-values < 0.01 considered to represent statistical significance.

Regarding plan quality, we evaluated the homogeneity index (HI; defined as [D_2%_ − D_98%_]/D_50%_); the 95% isodose conformity index (CI_95_; defined as V_95%_/V_PTV_), where V_95%_ is the volume covered by 95% of the prescribed dose and V_PTV_ is the PTV volume; and DVH parameters of the KBP_CONST_ and CMO plans [13, 28]. Additionally, the dose distributions of the KBP_CONST_ and CMO plans of the 15 patients were compared by two expert radiation oncologists. The dose distributions of the KBP_CONST_ were graded as superior, comparable or inferior to those of the CMOplan.

## RESULTS

The DVH parameters for KBP_ORIG_, KBP_CONST_ and CMO plans are summarized in Table 4. The mean PTV D_98%_ and D_2%_ for KBP_ORIG_ were 58.6 and 76.4 Gy, respectively. These values were significantly better than those of the KBP_CONST_ (56.7 and 78.1 Gy; both *P* < 0.001) and CMO plans (54.6 and 79.3 Gy; both *P* < 0.01). The mean D_2%_ of the PTV for KBP_CONST_ was significantly smaller than that of the CMO plans (*P* < 0.0001), whereas the D_98%_ and D_50%_ of the PTV for KBP_CONST_ were comparable to those of the CMO plans.

**Table 4:** DVH parameters for PTV and OARs, and MU and MCSv for KBP_CONST_, KBP_ORIG_ and CMO plans

	Parameter	(a) KBP_CONST_ (Gy)	(b) KBP_ORIG_ (Gy)	(c) CMO plans (Gy)	*P-*value
PTV	D_98%_	56.7 ± 2.5	58.6 ± 2.2	54.6 ± 3.9	a vs b: 0.0003
					b vs c: 0.004
	D_50%_	72.9 ± 0.4	71.5 ± 1.8	73.0 ± 0.4	NS
	D_2%_	78.1 ± 1.0	76.4 ± 0.9	79.3 ± 1.5	a vs b: 0.0005
					b vs c: <0.0001
					a vs c: <0.0001
Spinal cord	D_max_	47.7 ± 1.1	72.1 ± 4.1	47.3 ± 2.3	a vs b: <0.0001
					b vs c: <0.0001
Brainstem	D_max_	48.8 ± 1.5	65.8 ± 2.0	52.9 ± 4.4	a vs b: <0.0001
					b vs c: <0.0001
					a vs c: 0.0006
Ipsilateral parotid gland	D_med_	26.2 ± 11.1	44.4 ± 8.5	36.1 ± 13.4	a vs b: <0.0001
					b vs c: 0.003
					a vs c: 0.002
	D_mean_	31.9 ± 7.8	45.5 ± 6.1	38.7 ± 10.2	a vs b: <0.0001
					b vs c: 0.002
					a vs c: 0.006
Contralateral parotid gland	D_med_	22.5 ± 3.9	40.2 ± 7.0	22.2 ± 8.2	a vs b: <0.0001
					b vs c: <0.0001
	D_mean_	28.7 ± 4.4	42.6 ± 5.5	28.0 ± 6.6	a vs b: <0.0001
					b vs c: <0.0001
MU		490 ± 31	448 ± 28	561 ± 68	b vs c: 0.0001
					a vs c: 0.0001
MCSv		0.29 ± 0.02	0.32 ± 0.02	0.28 ± 0.03	a vs b: 0.0003
					b vs c: 0.0002

In terms of OARs, the mean D_max_ of spinal cord and brainstem for KBP_ORIG_ (72.1 and 65.8 Gy) were clinically unacceptable. The DVH parameters of KBP_ORIG_ did not meet our goal for all OARs (Table 2). Thus, the KBP_ORIG_ was deemed unacceptable for clinical use. In contrast, for KBP_CONST_, the mean D_max_ of spinal cord and the mean D_med_ and D_mean_ of contralateral parotid glands were comparable with those of the CMO plans; moreover, the mean D_max_ of brainstem (48.8 Gy) and mean D_med_ and D_mean_ of ipsilateral parotid glands (26.2 and 31.9 Gy, respectively) for KBP_CONST_ were significantly lower than those of the CMO plans. Thus, in terms of DVH parameters, KBP_CONST_ were comparable with the CMO plans. The MU values of the KBP_CONST_ and KBP_ORIG_ were significantly lower than those of the CMO plans (*P* < 0.001). MCSv of KBP_CONST_ were larger than those of CMO plans, although the difference was not statistically significant (Table 4).

HI and CI_95_ of KBP_CONST_ were also found to be comparable with those of the CMO plans. The dose distribution of the KBP_CONST_ and CMO plans for the 15 patients were subsequently compared for all CT slices. Discussions between two expert radiation oncologists regarding each of the 15 patients led to the conclusion that the KBP_CONST_ were superior to the CMO plans in seven cases (47%), comparable in six (40%) and inferior in two (13%).

**Fig. 1.**
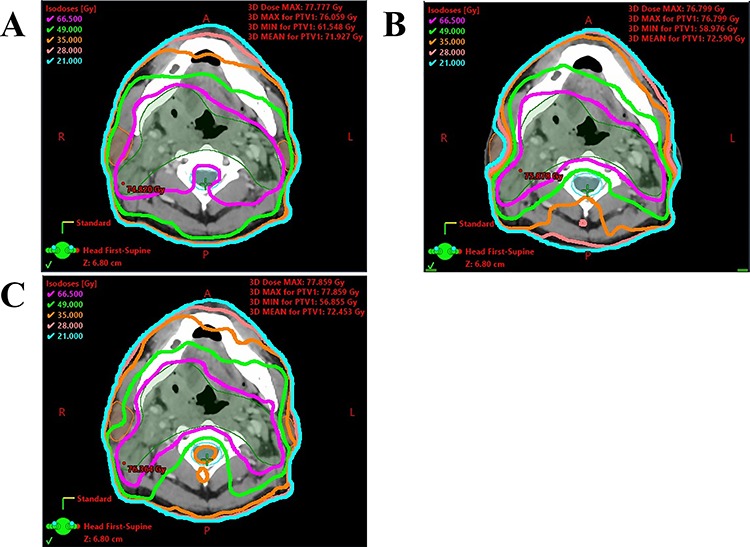
(**A**) KBP without manual objective constraints from a single optimization for right tonsil cancer, (**B**) KBP with manual objective constraints from a single optimization for the same patient, and (**C**) CMO plan for the same patient. Green line, 49 Gy iso-dose line; orange line, 35 Gy iso-doseline.

Figure 1 shows the dose distributions in a patient with right tonsil cancer. In the KBP_ORIG_ (Fig. 1A), the spinal cord was included in the high dose region; thus, the KBP_ORIG_ was clinically unacceptable. In terms of the KBP_CONST_ (Fig. 1B) and the CMO plan (Fig. 1C), there were no differences in PTV coverage and sparing of the spinal cord. In addition, the bilateral parotid glands were better spared by the KBP_CONST_ than by the CMO plan. In this particular patient, the D_med_ values of the ipsilateral and contralateral parotid glands for the CMO plan were 47.7 and 26.2 Gy, respectively. This patient’s clinical plan could not fully meet our goal in terms of D_med_ for parotid glands, although the planners accepted the plan finally after repeated re-optimization. In contrast, the D_med_ of the ipsilateral and contralateral parotid glands for KBP_CONST_ were 19.5 and 18.0 Gy, respectively. Thus, in this patient, the KBP_CONST_ was superior in quality to the CMOplan.

## DISCUSSION

The KBP_ORIG_ and KBP_CONST_ from a single optimization and CMO plans were compared. KBP_ORIG_ was found to be clinically unacceptable due to high doses to important OARs including neurological structures. Similarly, Chang *et al*. showed that half of their KBP for patients with NPC did not achieve their DVH goals for neurologic structures [11]. Kamima *et al*. reported that KBP could not achieve their study criteria for the spinal cord and brainstem without manual objective constraints from a single optimization [24]. On the other hand, in the present study, KBP_CONST_ from a single optimization was comparable or superior to CMO plans for most patients with head and neck cancer. The difference between KBP_ORIG_ and KBP_CONST_ was manual setting of objective constraints for OARs. Our study revealed the importance of adding objective constraints for KBP from a single optimization.

Hussein *et al*. demonstrated that KBP from a single optimization and using automatically generated objective constraints were acceptable in the pelvic region [13], because there are fewer OARs in the pelvic region than in the head and neck region. The anatomical complexity of the head and neck region hinders the achievement of DVH goals for pharyngeal cancer. Most investigators have used only automatically generated objective constraints for OARs when comparing DVH parameters between KBP and CMO plans [2, 4, 6, 7, 11, 13–15, 22, 23]. However, in the present study, the KBP from a single optimization with manual addition of objective constraints to PTV and OARs were comparable to or better than CMO plans in 87% of patients. These adopted objective constraints for OARs were found by trial and error to improve the worst CMO plan of the 55 patients in the training set. In addition, we set objective constraints to meet our goals, especially for neurological structures, by setting priority of spinal cord and brainstem strictly (Table 3). Only line objectives were used for constraints of OARs in KBP_ORIG_. Similarly, the previous studies described that line objectives were weak for important structures such as the spinal cord and brainstem [11, 24]. Thus, setting objective constraints manually was an important factor for the clinical application ofKBP.

In two of the 15 patients, the dose distribution of the KBP_CONST_ was clinically inferior to that of the CMO plans, although the DVH parameters of the PTV and OARs, HI and CI_95_ were comparable. As hot spots in the larynx were noted in the both cases, the plans were regarded as inferior to the CMO plans. Many investigators do not evaluate dose distributions clinically [2–4, 11, 13, 23]. Both comprehensive assessment of each plan by radiation oncologists and evaluation of DVH parameters were necessary for evaluation ofKBP.

In this study, KBP_CONST_ and KBP_ORIG_ resulted in a significant reduction in the number of MUs compared with CMO plans (*P* < 0.001). Kubo *et al*. stated that increased MUs in KBP for prostate cancer implied that KBP are more complicated to deliver than CMO plans [2]. However, the results of this study including MCSv suggest that KBP might reduce plan complexity when appropriate objective constraints wereused.

Finally, KBP_CONST_ could resolve the limitation of the time-consuming optimization process. Approximately 1–2 h were required to create each of the CMO plans with repeated re-optimization, whereas a single optimization of each KBP_CONST_ took only 15 min. In addition, KBP_CONST_ from a single optimization also reduced the dependence on the planners’ ability, meaning that less-experienced planners could produce good-quality plans [11, 29].

In conclusion, KBP_ORIG_ was found to be clinically unacceptable, while KBP_CONST_ from a single optimization was comparable with or superior to CMO plans for most patients with head and neck cancer. Manual addition of appropriate objective constraints improved both KBP plan quality and DVH parameters. KBP_CONST_ can overcome the limitation of the consuming lengthy optimization process and shows good potential for clinicaluse.
